# Tobacco exposure as a major modifier of oncologic outcomes in human papillomavirus (HPV) associated oropharyngeal squamous cell carcinoma

**DOI:** 10.1186/s12885-020-07427-7

**Published:** 2020-09-23

**Authors:** Hesham Elhalawani, Abdallah S. R. Mohamed, Baher Elgohari, Timothy A. Lin, Andrew G. Sikora, Stephen Y. Lai, Abdelrahman Abusaif, Jack Phan, William H. Morrison, G. Brandon Gunn, David I. Rosenthal, Adam S. Garden, Clifton D. Fuller, Vlad C. Sandulache

**Affiliations:** 1 Department of Radiation Oncology, The University of Texas MD Anderson Cancer Center, 1515 Holcombe Blvd, 0097, FCT10.6002, Houston, TX 77030 USA; 2 MD Anderson Cancer Center UTHealth Graduate School of Biomedical Sciences, The University of Texas MD Anderson Cancer Center, Houston, TX USA; 3 ENT Section, Operative Care Line, Michael E. DeBakey Veterans Affairs Medical Center, Houston, TX USA; 4 Bobby R. Alford Department of Otolaryngology Head and Neck Surgery, Baylor College of Medicine, One Baylor Plaza, MS: NA102, Houston, TX 77030 USA; 5 Department of Head and Neck Surgery, University of Texas MD Anderson Cancer Center, Houston, TX USA; 6 Department of Biostatistics, The University of Texas MD Anderson Cancer Center, Houston, TX USA; 7 Medical Physics Program, The University of Texas Graduate School of Biomedical Sciences, Houston, TX USA; 8 Center for Translational Research on Inflammatory Diseases, Michael E. DeBakey Veterans Affairs Medical Center, Houston, TX USA

**Keywords:** Oropharyngeal carcinoma, Radiotherapy, Tobacco, Human papillomavirus

## Abstract

**Background:**

The incidence of oropharyngeal squamous cell carcinoma (OPSCC) in the US is rapidly increasing, driven largely by the epidemic of human papillomavirus (HPV)-mediated OPSCC. Although survival for patients with HPV mediated OPSCC (HPV+ OPSCC) is generally better than that of patients with non-virally mediated OPSCC, this effect is not uniform. We hypothesized that tobacco exposure remains a critical modifier of survival for HPV+ OPSCC patients.

**Methods:**

We conducted a retrospective analysis of 611 OPSCC patients with concordant p16 and HPV testing treated at a single institute (2002–2013). Survival analysis was performed using Kaplan-Meier analysis and Cox regression. Recursive partitioning analysis (RPA) was used to define tobacco exposure associated with survival (*p* < 0.05).

**Results:**

Tobacco exposure impacted overall survival (OS) for HPV+ patients on univariate and multivariate analysis (*p* = 0.002, *p* = 0.003 respectively). RPA identified 30 pack-years (PY) as a threshold at which survival became significantly worse in HPV+ patients. OS and disease-free survival (DFS) for HPV+ > 30 PY patients didn’t differ significantly from HPV- patients (*p* = 0.72, *p* = 0.27, respectively). HPV+ > 30 PY patients had substantially lower 5-year OS when compared to their ≤30 PYs counterparts: 78.4% vs 91.6%; *p* = 0.03, 76% vs 88.3%; *p* = 0.07, and 52.3% vs 74%; *p* = 0.05, for stages I, II, and III (AJCC 8th Edition Manual), respectively.

**Conclusions:**

Tobacco exposure can eliminate the survival benefit associated with HPV+ status in OPSCC patients. Until this effect can be clearly quantified using prospective datasets, de-escalation of treatment for HPV + OPSCC smokers should be avoided.

## Highlights

- Tobacco remains a critical driver of survival and treatment response in HPV+ OPC
- Clinical trials are investigating treatment deintensification in advanced HPV+ OPC
- Current efforts to consider tobacco exposure in this context should be encouraged

## Background

The human papillomavirus (HPV) has transformed the landscape of cancer diagnosis and treatment. Oropharyngeal squamous cell carcinoma (OPSCC), a disease traditionally associated with tobacco and alcohol exposure, is now overwhelmingly a disease associated with HPV [1–4]. This change in the epidemiology of the disease has resulted in dramatic improvements in treatment efficacy along with disease specific and overall survival [1–6]. The 8th Edition of the American Joint Committee on Cancer (AJCC) Staging Manual dramatically downstages HPV mediated (HPV+) OPSCC tumors relative to the prior edition [7]. Specifically, a significant fraction of HPV+ OPSCC tumors previously staged as stage III-IV has now been down-staged to stage I-II, consistent with the improved survival for HPV+ OPSCC patients compared to their HPV negative (HPV-) counterparts [7].

In 2010, Ang et al. identified an interaction between HPV status and tobacco exposure in OPSCC patients and defined “low-risk” and “intermediate-risk” tumors based on differential survival [1]. The principal distinction between the two risk categories was a reduced survival in patients who were smokers. Although this observation has been confirmed in subsequent studies, the interaction between HPV and cigarette smoking remains poorly characterized both from a biological perspective and with respect to clinical outcomes [8]. Tobacco exposure is generally expressed as a function of volume and time (1 pack-year (PY) = 1 pack of cigarettes per day for 1 year). Ang et al. set a threshold of 10 PY to define a “smoker” [1]. Although sufficient to impact survival, this threshold has not been fully validated in the context of HPV+ OPSCC. For certain patient populations a threshold of 10 PY results in defining nearly 90% of the patient population as smokers [8]. Studies of Veterans with HPV+ OPSCC have quantified tobacco exposure as high as 150 PY [8]. Although smoking rates have decreased over the last two decades, evidence suggests that tobacco exposure remains a significant consideration for OPSCC patients throughout the United States and varies widely based on race/ethnicity and socioeconomic strata [3, 9].

The recent changes to the AJCC staging system for OPSCC create an urgent need to better define the potential impact of tobacco exposure on clinical outcomes for HPV+ OPSCC in order to generate appropriate pre-treatment risk stratification and provide appropriate patient counseling. Furthermore, given continued efforts at treatment de-escalation for HPV+ OPSCC tumors, it is critical to determine whether a subset of patients may be at risk for inappropriate de-escalation. We hypothesized that tobacco exposure has a significant negative impact on HPV+ OPSCC patient survival and sought to test this hypothesis in a large single-institution cohort of patients with robust tumor biological data (concordant p16/HPV testing status) who received radiation-based treatment.

## Methods

### Patients

Following approval by the University of Texas MD Anderson Cancer Center institutional review board, we reviewed 1171 patients with a primary diagnosis of OPSCC who underwent definitive non-surgical treatment between 2002 and 2013. This end date was chosen to allow for a minimum follow up period of 5 years. Patients with recurrent disease, previous oncologic treatment, and/or lost to follow-up were excluded from the analysis. Demographics, tobacco usage, and clinical-pathologic history were comprehensively reviewed through the institutional electronic medical record. Cancer staging was conducted according to the American Joint Commission on Cancer Staging Manual (7th and 8th Editions). Tobacco exposure was obtained from the medical record in the form of PY defined as: 1 PY = 1 pack of cigarettes/day for 1 year. Supplementary Figure 1 represents a CONSORT flow diagram showing inclusion and exclusion criteria for the study patients.

#### HPV determination

Tumors were tested for HPV by use of the in situ hybridization (ISH)-catalyzed signal amplification method for biotinylated probes and for p16 protein expression via immunohistochemistry (IHC) consistent with clinical practice at our institution. Only patients with concordant p16 and HPV testing results were included in this analysis [1, 8].

### Treatment

The treatment strategy for individual patients was determined by discussion at the institutional multidisciplinary tumor conference. Surgically treated patients were excluded from this analysis. All patients were treated using intensity-modulated radiotherapy (IMRT) using previously described protocols [10, 11]. Generally, we use IMRT to treat the primary tumor and the upper neck nodal disease matched to an anteroposterior low anterior neck field with a larynx midline block (IMRT split-field technique). Whole-field IMRT was used for junctional tumors to avoid under-dosing. Small volume primary tumors were usually prescribed up to 66 Gy, while more advanced tumors were prescribed up to 70–72 Gy. Radiation was delivered using 6-MV photons linear accelerators. Decisions of systemic therapy addition to IMRT were individualized based on the disease burden as well as associated medical comorbidities and performance status. Concurrent chemo/IMRT was prescribed to patients with advanced primary tumor and/or bulky lymph node metastasis, while induction chemotherapy was assigned for patients with high risk of distant recurrence (i.e. advanced N-stage) [12].

### Comorbidity assessment

We used the Charlson Comorbidity Index (CCI) to assess pre-treatment patient comorbidity status [13]. The CCI has been validated in head and neck cancer populations by Singh et al. who described its utility and ease of use in the setting of retrospective studies [14]. We also calculated the age-adjusted CCI by adding one point to the baseline CCI score for every additional decade over the age of 40 [15].

### Study endpoints and statistical analysis

The outcomes of interest included: ‘Loco-regional control (LRC)’ defined as time from date of completion of treatment to date of diagnosis of local and/or regional recurrence; ‘Freedom from distant metastasis (FDM)’ defined as time from date of completion of treatment to date of diagnosis of malignant metastasis to distant body organ; ‘Disease-free survival (DFS)’ defined as time from date of completion of treatment to date of diagnosis of loco-regional and/or distant recurrence (i.e. whichever occurred initially); and ‘Overall survival (OS)’ defined as time from date of completion of treatment until death or last recorded follow-up. Chi-square tests were used to compare the categorical variables (i.e. sex, race, T-classification, N-classification, comorbidities etc.) between the p16+/HPV+ (abbreviated to HPV+) versus p16−/HPV- (abbreviated to HPV-) cohorts. Survival analysis was performed using Kaplan-Meier analysis (log-rank test). Uni- and multi-variable survival analyses were performed using Cox regression. Recursive partitioning analysis (RPA) was used to quantify a threshold for tobacco exposure significantly associated with overall survival (*p* < 0.05). For multivariable analysis, we tested the prognostic impact of the AJCC staging system and tobacco exposure in HPV+ patients compared with a baseline model of standard clinical variables. The baseline model included age, sex, AJCC 7th edition, chemotherapy sequence, and total EBRT dose. We then compared the alternative models using Bayesian information criteria [16] (BIC). A lower BIC indicates improved model performance and parsimony, using the BIC evidence grades presented by Raftery [17] with the posterior probability of superiority of a lower BIC model, where a BIC decrease of < 2 is considered “Weak” (representing a 50–75% posterior probability of being superior model), 2–6 denoted “Positive” (posterior probability of 75–95%), 6–10 as “Strong” (posterior probability of > 95%), and > 10, “Very strong” (posterior probability > 99%). We analyzed the competing risk of failure and death using Weibull parametric fitting of cause of failure and death, respectively, as a competing risk variable for uncensored data. Statistical analysis was performed using JMP Pro statistical software (version 11.2.0; SAS Institute Inc., Cary, NC).

## Results

### Patients

A total of 611 patients were included in the analysis. The majority (89%) were HPV+ and half of these patients reported 0 PY history of tobacco exposure whereas only 20% of the HPV- patients were non-smokers. Approximately one-third of HPV+ smokers reported heavy exposure (i.e. > 30 PY history) compared with approximately one-half of HPV- smokers. The details of tobacco exposure in PY for both cohorts are shown in Supplementary Figure 2. The HPV+ cohort included more males than the HPV- cohort (87% vs 68%, *p* < 0.0001). Subsite distribution was comparable between the cohorts. One hundred twenty six patients (21%) had at least one pretreatment comorbid condition. The most common comorbid condition was diabetes mellitus (*n* = 53 patients, 9%), followed by cardiovascular conditions (*n* = 30 patients, 5%) and respiratory conditions (*n* = 27 patients, 4%). There was no statistically significant difference in CCI score between the HPV+ and HPV- cohorts (Table 1). Most patients in both cohorts presented with early T-classification (T1–2) tumors while nodal stage varied significantly with the most frequent N-classification shifting from N2 (AJCC 7th edition) to N1 (AJCC 8th edition) for the HPV+ cohort as detailed in Table 1. The treatment regimens did not vary significantly between the cohorts.

**Table 1:** Patients, tumor and treatment characteristics

Variable	p16+/HPV+ N (%)	p16−/HPV- N (%)	***p***-value
	*N* = 546 (89.4%)	*N* = 65 (10.6%)	
Age			*p* = 0.84
< 50	86 (15.8)	11 (16.9)	
50- < 60	231 (42.3)	25 (38.5)	
≥ 60	229 (41.9)	29 (44.6)	
Sex			p < 0.0001*
Male	473 (86.6)	44 (67.7)	
Female	73 (13.4)	21 (32.3)	
Race			*p* = 0.08
White	505 (92.5)	56 (86.2)	
Non-white	41 (7.5)	9 (13.8)	
Cancer subsite of origin			p = 0.8
Base of tongue	250 (45.8)	27 (41.5)	
Tonsil	252 (46.2)	26 (40)	
Other	44 (8)	12 (18.5)	
CCI			p = 0.7
0 (No comorbidity)	436 (80)	49 (75)	
≥ 1 (At least one comorbidity)	110 (20)	16 (25)	
T-category			*p* = 0.09
T1	168 (30.8)	17 (26.2)	
T2	211 (38.6)	18 (27.6)	
T3	98 (17.9)	15 (23.1)	
T4	69 (12.7)	15 (23.1)	
N-category			p < 0.0001*
N0	39 (7.1)	8 (12.3)	
N1	373 (68.3)	10 (15.4)	
N2	121 (22.2)	45 (69.2)	
N3	13 (2.4)	2 (3.1)	
AJCC (8th edition)			p < 0.0001*
I	316 (57.9)	1 (1.5)	
II	149 (27.3)	4 (6.2)	
III	81 (14.8)	13 (20.0)	
IV	0	47 (72.3)	
Smoking status at diagnosis			< 0.0001
Current smoker	101 (18.5)	25 (38.46)	
Former smoker	208 (38.1)	28 (43.08)	
Never smoker	249 (40.75)	12 (18.46)	
Tobacco exposure (PY**)**			p < 0.0001*
0	277 (50.7)	13 (20)	
> 0–10	87 (15.9)	6 (9.2)	
> 10–20	50 (9.2)	8 (12.3)	
> 20–30	46 (8.4)	11 (17)	
> 30	86 (15.8)	27 (41.5)	
Tobacco exposure per AJCC (8th edition) stage			p < 0.0001
I/pack-years < 30	263 (48.2)	1 (1.5)	
I/pack-years ≥30	53 (9.7)	0	
II/pack-years < 30	113 (20.7)	1 (1.5)	
II/pack-years ≥30	36 (6.6)	3 (4.6)	
III/pack-years < 30	59 (10.8)	4 (6.2)	
III/pack-years ≥30	22 (4)	9 (13.8)	
IV/pack-years < 30	0	27 (41.6)	
IV/pack-years ≥30	0	20 (30.8)	
Chemotherapy (Cth) sequence			*p* = 0.94
No Cth	99 (18.1)	12 (18.5)	
Induction Cth (IC) only	82 (15.0)	8 (12.2)	
Concurrent Cth (CC) only	210 (38.5)	25 (38.5)	
IC + CC	155 (28.4)	20 (30.8)	

*Abbreviations*: *EBRT* external beam radiation therapy, *IC* induction chemotherapy, *CEBRT* concurrent chemotherapy + EBRT. * indicates *p* < 0.05 when comparing the HPV+ and HPV- groups

### HPV status impacts OPSCC patient survival

**Fig. 1:**
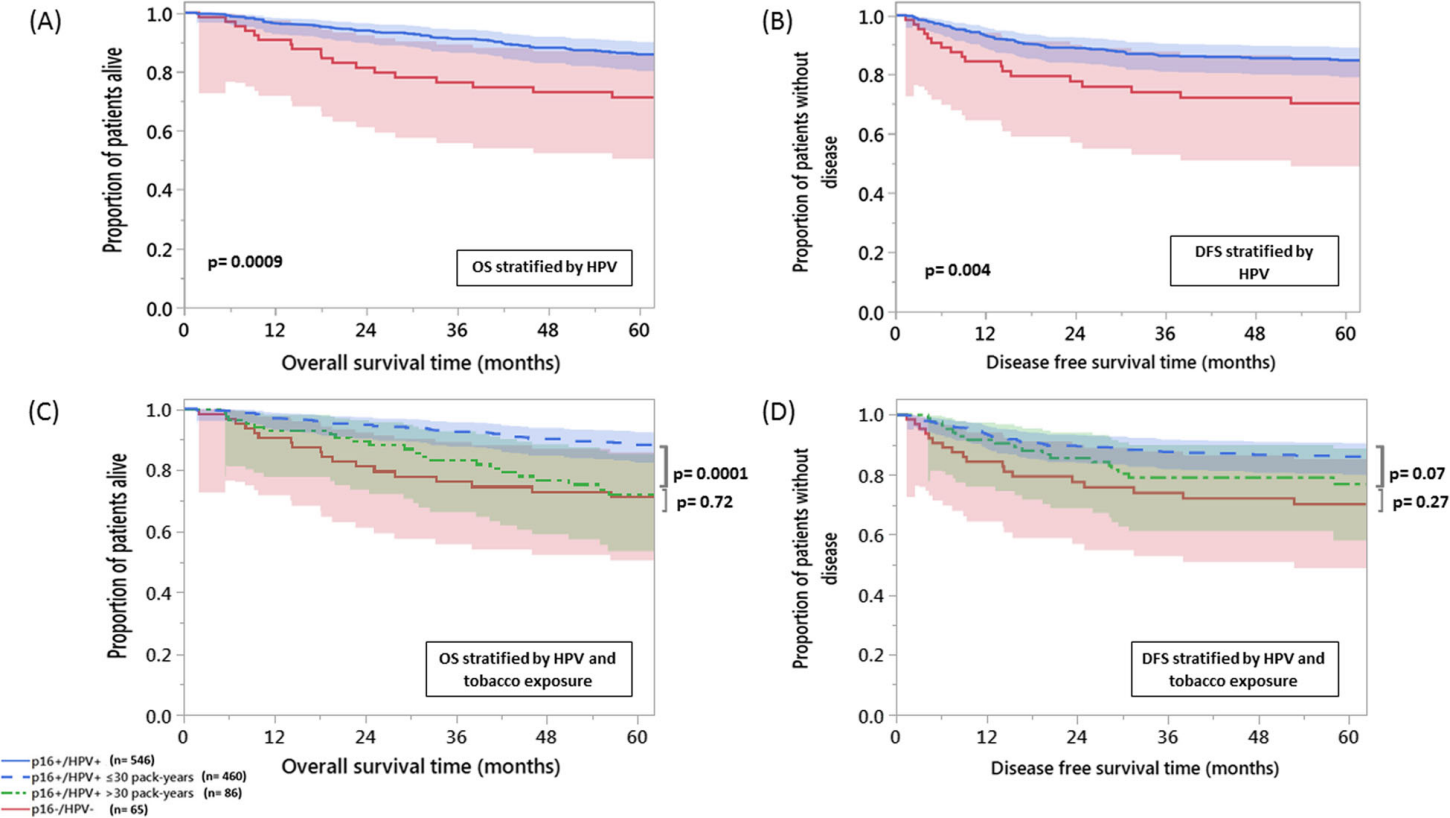
Heavy tobacco exposure decreases overall and disease free survival for patients with HPV + OPC. Kaplan-Meier curves of overall survival ‘OS’ (**a**) and disease-free survival ‘DFS’ (**b**) for the entire cohort stratified by human papillomavirus (HPV) status; and OS (**c**) and DFS (**d**) for the entire cohort stratified by HPV status and tobacco exposure. Kaplan-Meier survival curves confidence intervals are represented by shades of the corresponding group color

The 5-year actuarial LRC, FDM, DFS, and OS rates for the entire cohort were 88.9, 91.3, 83.3, and 84.3% respectively. The 5-year outcomes for the HPV+ cohort were favorable compared with the HPV- cohort for all studied endpoints except FDM (OS 85.8% vs 71.2%, *p* = 0.0009; DFS 84.7% vs 70.2%, *p* = 0.004; LRC 89.8% vs 81.2%, *p* = 0.040; FDM 91.7% vs 88.3%, *p* = 0.44) (Fig. 1, Supplementary Figure 3).

### Tobacco exposure impacts OPSCC patient survival

Univariable analysis demonstrated that tobacco exposure (quantified in PY) was significantly associated with OS in both the HPV+ and HPV- cohorts (*p* = 0.002 and 0.0006, respectively). Subsequent RPA identified 30 PY as the cut-off threshold for differential OS risk; the resulting binary risk groups (PY ≤30 and PY > 30) were then integrated into the final analysis. Smokers with > 30 PY of exposure were shown to have more than double the hazard of death in both the HPV+ (HR = 2.6, 95%CI = 1.5–4.2, *p* = 0.0006) and HPV- (HR = 2.7, 95%CI = 1.1–7.5, *p* = 0.04) cohorts as compared to patients with ≤30 PY of exposure. The following clinical variables were also significantly associated with OS in the HPV+ cohort: T-classification, AJCC stage (8th edition), CCI, chemotherapy sequence and total radiation dose. In the HPV- cohort, T-classification was the only additional clinical variable with significant association with OS (Supplementary Table 1). Using multivariable analysis for HPV+ patients CCI, PY binary smoking index and AJCC 8th edition were the remaining significant variables associated with OS. Among HPV- patients, however, none of the examined variable remained significant in multivariable analysis (Supplementary Table 1).

Patients in the HPV+ cohort with tobacco exposure > 30 PY had decreased 5-year OS (72.1% vs 88.3%, *p* = 0.0001) and 5-year DFS (76.9% vs 86.1%, *p* = 0.07) compared to patients with tobacco exposure ≤30 PY (Fig. 1). While 5-year LRC was worse in the HPV+ > 30 PY cohort (81.7% vs 91.2%, *p* = 0.02), 5-year FDM did not demonstrate significant differences between the two subsets (91.2% vs 91.8%, *p* = 0.39) (Supplementary Figure 4).

Overall survival and DFS Kaplan-Meier survival probability estimates at 5 years did not statistically differ for the HPV+ > 30 PY and HPV- cohorts (72.1% vs 71.2%, *p* = 0.72; 76.9% vs 70.2%, *p* = 0.27, respectively). Smoking status at diagnosis did not significantly correlate to OS or DFS in HPV+ patients on univariable analysis. When patients in the HPV+ cohort with tobacco exposure > 30 PY were stratified into current and former smokers (*n* = 43 each), OS and DFS Kaplan-Meier survival probability estimates at 5 years still did not statistically differ between the two subgroups.

**Fig. 2:**
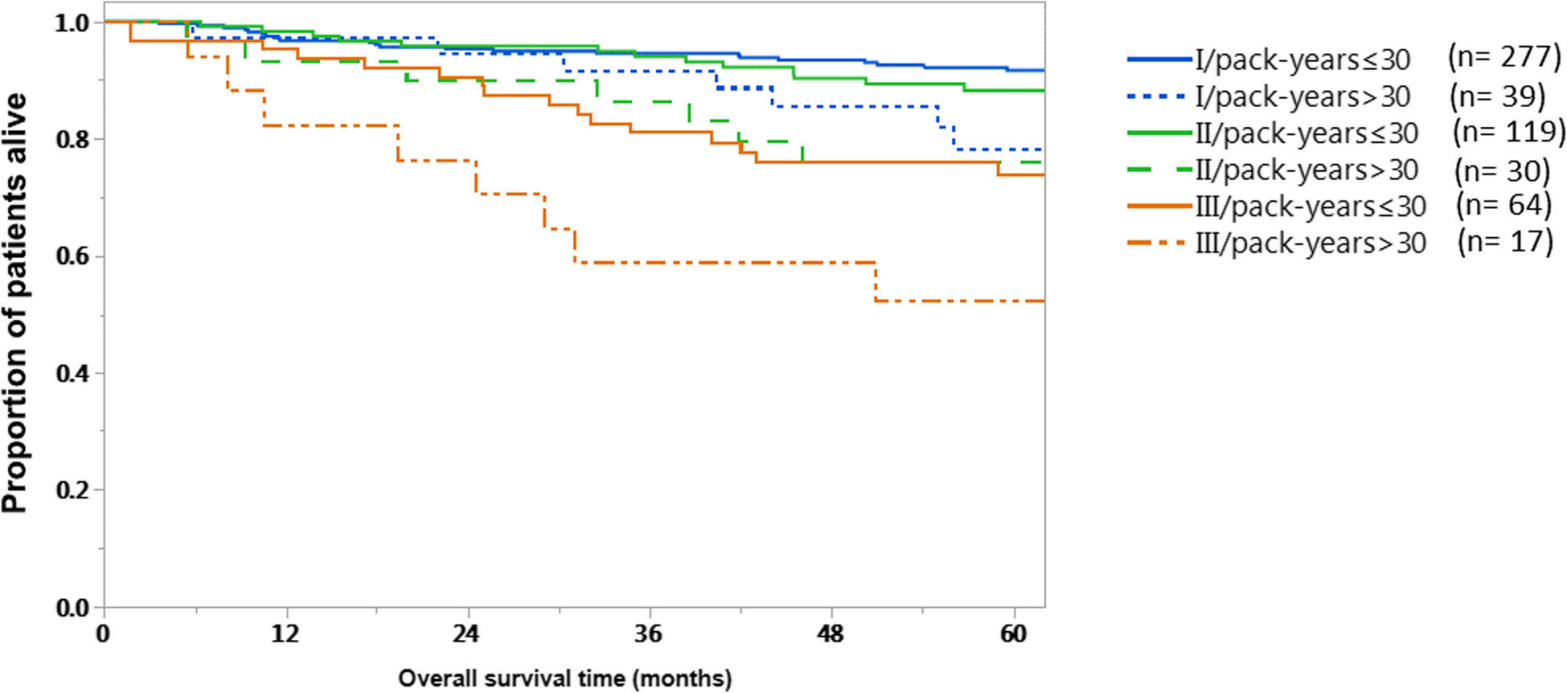
Heavy tobacco exposure decreases HPV + OPC overall survival across AJCC stages. Kaplan-Meier overall survival (OS) curves for HPV + OPSCC AJCC (8th edition) stages I-III. Solid lines denote survival curves for each individual stage in the absence of heavy tobacco exposure; dotted lines denote survival for each individual stage in the presence of heavy tobacco exposure

We stratified stage I-III (AJCC 8th edition) HPV+ patients into low (≤30 PY) and high (> 30 PY) tobacco exposure (Fig. 2). Patients with higher tobacco exposure in each stage showed worse 5-year OS compared to their counterparts. The 5-year OS for HPV+ patients with > 30 vs ≤30 PY was 78.4% vs 91.6%; *p* = 0.03, 76% vs 88.3%; *p* = 0.07, and 52.3% vs 74%; *p* = 0.05, for stages I, II, and III, respectively.

On multivariable analysis, AJCC 8th edition markedly improved the OS model performance over the baseline model when added instead of the 7th edition (i.e. BIC decreased by 11). Further addition of the tobacco risk grouping achieved the best OS model performance (BIC decreased by 13 over the baseline and 2 over the AJCC 8th edition models, respectively). Uni- and multi-variable analysis for DFS showed no correlation between tobacco exposure and DFS in either the HPV+ or HPV- cohorts. While chemotherapy sequence, total EBRT dose, T- and N-classifications, and AJCC stage (8th edition) were associated with DFS in the HPV+ cohort on univariable analysis, only AJCC stage (8th edition) retained significant association with DFS when combined into a multivariable model. On the other hand, DFS in HPV- OPSCC subset was significantly associated with age at diagnosis and T-classification (on univariable analysis), and total EBRT dose (on both analyses) (Supplementary Table 2).

### Competing risk analysis

**Fig. 3:**
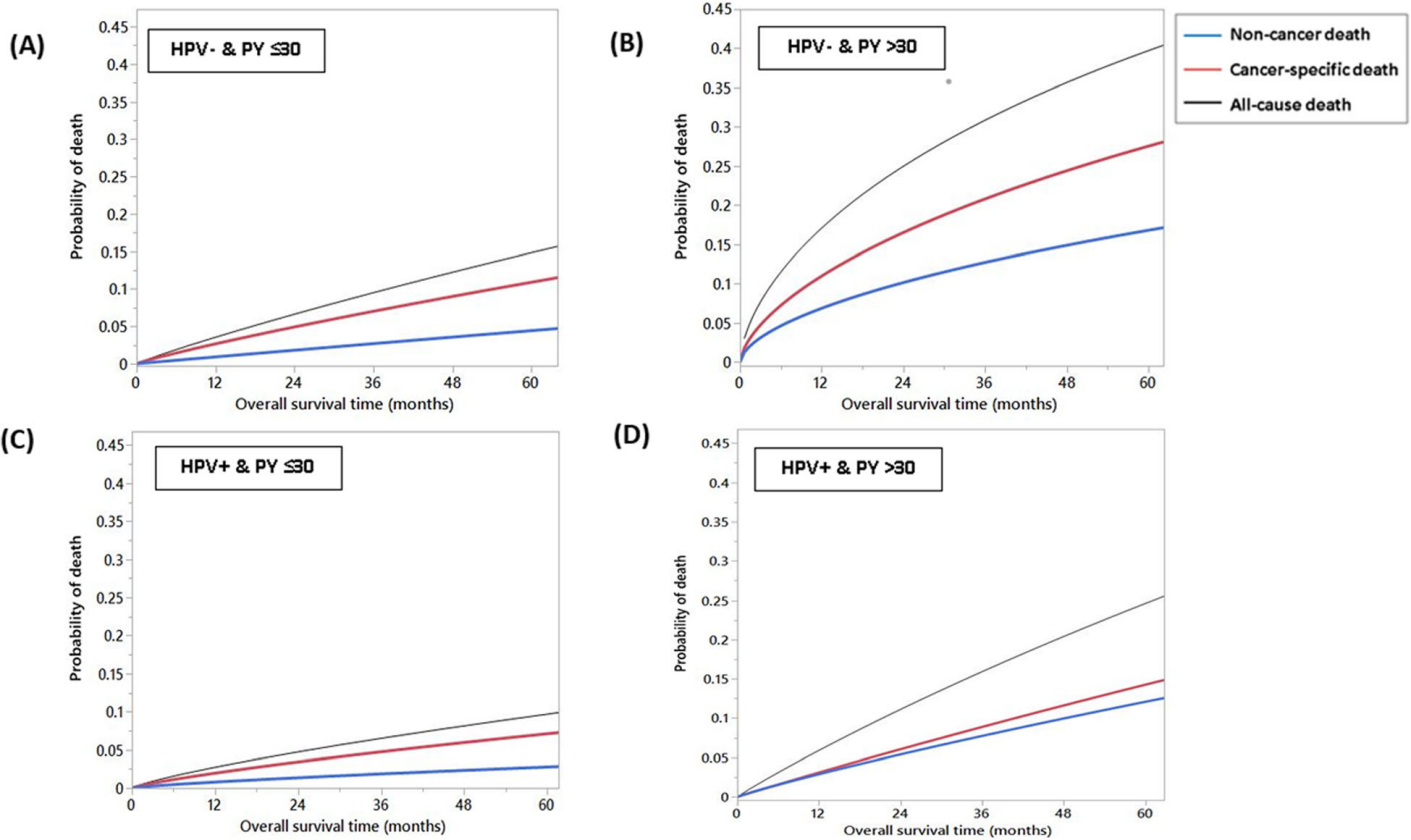
Competing risks models for causes of death in subpopulations stratified by human papillomavirus (HPV) and tobacco exposure: (**a**) HPV- & pack-years (PY) ≤30; (**b**) HPV- & PY > 30; (**c**) HPV+ & PY ≤30; and (**d**) HPV+ & PY > 30. Lines are curves fitting all cause death events (black), cancer-specific death events (red) and non-cancer death events (blue)

**Fig. 4:**
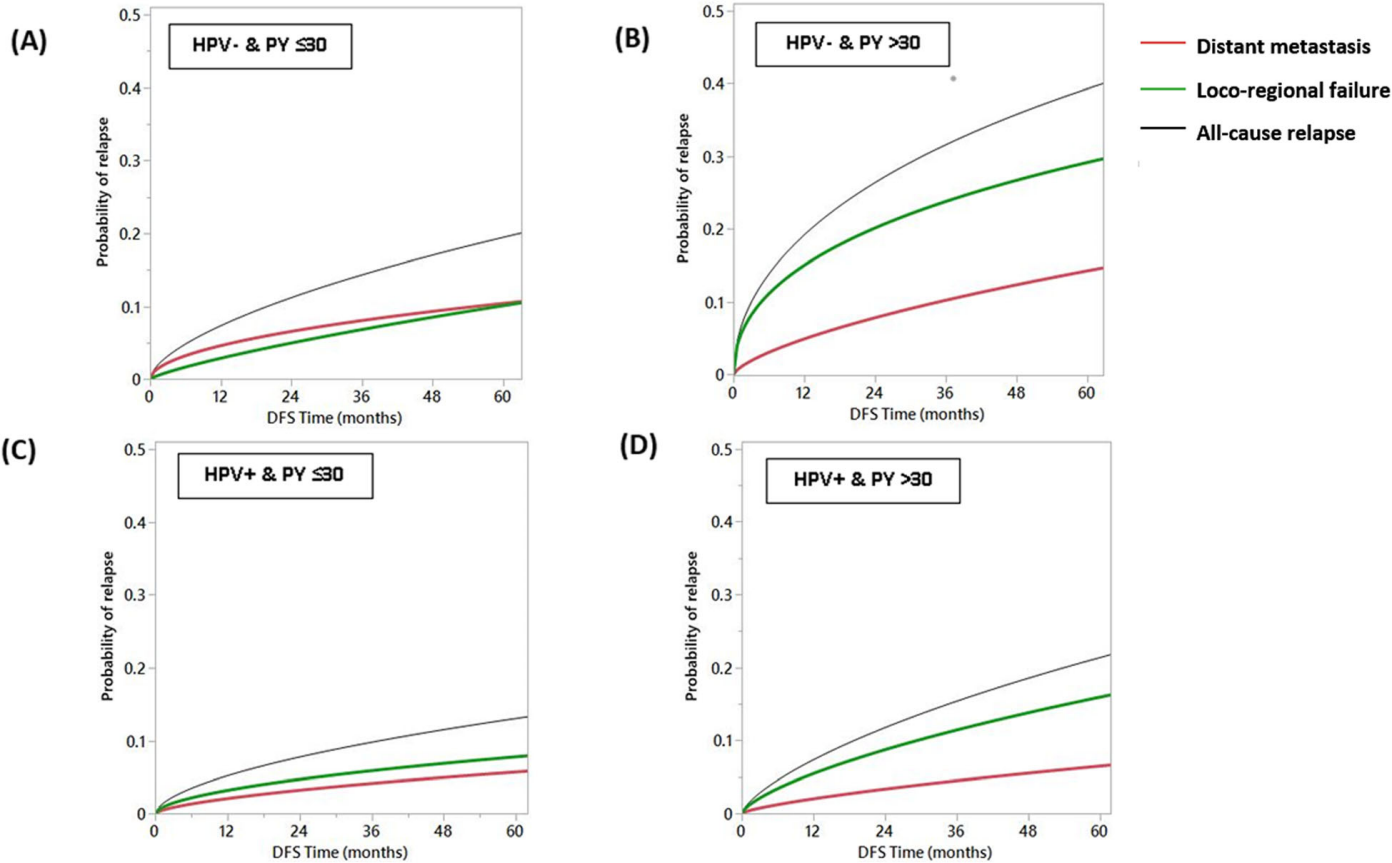
Competing risks models for causes of relapse in subpopulations stratified by human papillomavirus (HPV) and tobacco exposure: (**a**) HPV- & pack-years (PY) ≤30; (**b**) HPV- & PY > 30; (**c**) HPV+ & PY ≤30; and (**d**) HPV+ & PY > 30. Lines are curves fitting all cause relapse events (black), loco-regional relapse events (green) and distant metastasis events (red)

A competing risk analysis of causes of death stratified by p16/HPV status and tobacco exposure demonstrated that index cancer-specific deaths were the predominant cause of death in the HPV- cohort independent of tobacco exposure status and in the HPV+ cohort with tobacco exposure≤30 PY as shown in Fig. 3a, b, and c, respectively. However, the HPV+ cohort with tobacco exposure > 30 PY had a higher risk of non-cancer deaths compared to other three subgroups (Fig. 3d). Furthermore, competing risk of the mode of failure in all the four OPSCC patient subsets revealed a much higher probability of loco-regional failure in patients with high (> 30 PY) tobacco exposure as compared to the lower tobacco exposure subgroups, regardless of the p16/HPV status. Compared to failures attributed to distant metastases, patients with high tobacco exposure have double the probability of developing loco-regional failure at 5-years (Fig. 4).

Given the potential impact of heavy tobacco exposure on non-cancer related mortality, we performed an additional analysis of this datapoint in the context of our patient cohort. Among patients with PY ≥ 30, 66 and 59% had no pre-treatment comorbidities (CCI = 0) in the HPV+ and HPV- cohorts, respectively. Distribution of comorbidities within the HPV+ cohort were as follows. Among patients with tobacco exposure < 30 PY, 1% had respiratory comorbidities, 8% had endocrine comorbidities and 4% had cardiovascular comorbidities. Among patients with tobacco exposure ≥30 pack-years 9% had respiratory comorbidities, 10% had endocrine comorbidities and 7% had cardiovascular comorbidities. Distribution of comorbidities within the HPV- cohort were as follows. Among patients with tobacco exposure < 30 PY, 0% had respiratory comorbidities, 5% had endocrine comorbidities and 5% had cardiovascular comorbidities. Among patients with tobacco exposure ≥30 PY 6% had respiratory comorbidities, 11% had endocrine comorbidities and 15% had cardiovascular comorbidities. For HPV+ patients higher CCI score (≥1) and higher PY history (≥30) were independently associated with higher non-cancer related mortality, both with more than triple the hazard (HR = 3.6, 95%CI = 1.5–8.5, *p* = 0.004 for CCI ≥ 1 and HR = 3.3, 95%CI = 1.4–7.6, *p* = 0.008 for PY ≥ 30). For HPV- patients none of the examined variables were significantly associated with non-cancer mortality (likely due to relatively lower sample size).

## Discussion

OPSCC incidence is rising at an alarming rate in the United States [2, 3, 18, 19]. The most recent analysis completed in 2018 demonstrated a persistently low rate of HPV preventive vaccination among the US population [20]. It is, therefore, reasonable to expect that the current increase in OPSCC incidence secondary to HPV is likely to continue for at least the next few decades, especially in North and South America, Central, Eastern, and Northern Europe [21]. As the OPSCC patient population is expected to increase, it is critical to improve our understanding of how disease biology interacts with and/or determines treatment response [2, 3, 8, 18, 19]. This requires not only an improved understanding of HPV-mediated effects on tumorigenesis and treatment response, but also a better understanding of the interaction between HPV exposure and other OPSCC risk factors such as tobacco exposure.

Despite a continued decrease in prevalence over the last half century, tobacco use remains associated with 4 out of 5 leading causes of death in the US. The interplay among smoking, HPV infection, other risk factors, and carcinogenesis is complex and multifactorial [22]. Increasing tobacco exposure has been linked to greater risk of oral HPV infection [23–25]. The interaction between tobacco exposure and HPV infection in OPSCC carcinogenesis and whether the risk of HPV-mediated OPSCC is higher or lower among smokers have been a matter of ongoing debate [26–28]. Our data in the US Veteran population indicate that tobacco exposure is nearly ubiquitous and that approximately 75% of new HPV+ OPSCC diagnoses occur in patients with > 10 pack-year history of tobacco exposure [8]. Similar data have been reported for other patient populations including indigent, uninsured and underinsured patients [29, 30].

The 8th Edition of the AJCC staging manual recognized the more favorable prognosis of patients with HPV-mediated OPSCC. As such, the current manual down-stages patients with what was historically regarded as locally advanced disease. However, while recognizing that patients with HPV-mediated disease have better prognoses, it has also been recognized that current and former smokers tend to have worse survival rates compared to non-smokers [7]. The AJCC was challenged with incorporating smoking into the staging, which was later described in the manual as follows: “the role of tobacco as a negative prognostic factor is well established. However, exactly how this could be codified in the staging system is less clear” [31]. Even prior to the AJCC 8th Edition era, similar challenges were reported when smoking was introduced into the prognostic framework. The model generated by Huang et al. which included smoking appeared robust for Stages I and II (by criteria used for 8th edition) at a threshold of 20 PY but did not hold for Stage III patients. That was attributed to the detrimental influence of age in the model, potentially related to inability to tolerate intensive treatment for these anatomically more extensive HPV+ lesions (T4 and N3) [32]. Our data strongly suggest that, although survival is greatly impacted by HPV status, tobacco exposure also plays a very important role. This has been strikingly demonstrated by a poorer survival in both HPV+ and HPV- heavy smokers (i.e. more than double the hazard of death) compared to < 30 PY smokers. This suggests that not all HPV+ OPSCC tumors should be expected to demonstrate the same excellent outcomes we have come to expect. Perhaps most concerning is the significant survival decrease in patients with heavy tobacco exposure. For overall survival, tobacco exposure erases the favorable survival impact of HPV positivity, generating an absolute survival decrement of approximately 16%. As a point of reference, the absolute survival benefit for the addition of chemotherapy to radiation was only ~ 8%, based on the most recent MACH-NC meta-analysis [33]. We recently updated survival for patients with oral cavity SCC, and identified the relative effect size for nodal metastasis at ~ 15% and extra-nodal extension at ~ 20% [34]. These data place the impact of tobacco exposure within the range of other treatment modifying clinical-pathologic parameters.

Furthermore, our results showed that the impact of heavy tobacco exposure is alarmingly impacting the outcomes of even early stage disease as defined by the newest edition of the AJCC staging system. Our results showed that the proposed tobacco exposure cutoff of 30 PY clearly stratified patients at each AJCC (8th edition) stage in terms of overall survival outcomes. However, statistical significance was only reached in stage I, probably because of smaller numbers of patients with more advanced disease (i.e. ~ 60% of the whole cohort was categorized as stage I). Nonetheless, heavy smokers with stage I or II disease had 5-year outcomes that were approximating or -in some cases- even worse than outcomes of patients with lower smoking index and more advanced disease stage. This observation must be considered in the inclusion criteria for future dose de-escalation studies in early stage HPV+ OPSCC. In agreement with these results, a recent study by Vawda et al. [35] has demonstrated that higher intensity of smoking exposure was associated with poorer outcomes in a cohort of exclusively HPV+ oropharyngeal cancer patients treated with primary radiation or surgery. The study, however, lacked the comparison with an HPV- cohort. As a result, the relative effect size of tobacco exposure in the HPV+ OPSCC population remains only partially contextualized. Our findings track closely with those of this recently published data. Moreover, our data indicate that HPV+ patients with smoking index above 30 have surprisingly comparable outcomes to HPV- patients, highlighting the importance of considering this very important risk factor in the treatment decision making process.

In the meantime, the putative impact of smoking on cancer-specific mortality in HPV+ OPSCC cannot be easily interpreted given the known adverse effects of smoking on general comorbidity and interaction with co-existing risk factors (i.e. alcohol consumption) [36, 37]. Our competing risk analysis indicated that the worse survival outcomes of HPV+ heavy smokers were attributable to two main factors; the increased risk of locoregional failure that leads to more cancer related deaths as well as the overall increased risk of non-cancer related deaths compared with smokers below the identified threshold (Figs. 3 and 4). That’s to say HPV+ heavy smokers who don’t die of smoking-related comorbidities (the dominant detrimental effect of smoking per Fig. 3d) will more probably die of loco-regional failure (Fig. 4d).

Although large, this is a single institution patient cohort and as such our findings must be validated in additional patient cohorts. It is also important to note, that our outcomes for HPV- patients in this series are dramatically better than historical data even from our own institution [1, 2, 6]. This, combined with the relative small size of the HPV- cohort may confound the comparison between the 3 groups outlined in Fig. 1. Moreover, chemotherapy, despite being associated by multiple randomized controlled trials with better treatment outcomes, was not shown to be an independent prognostic factor even in HPV- subpopulation where more benefit from chemotherapy would be expected [38]. This can be attributed in part to the retrospective nature of the study where treatment decisions followed the institutional multidisciplinary protocol. That’s to say, patients with early stage disease, with more favorable prognosis, received no chemotherapy in contrast to the more advanced HPV- tumors which still showed worse outcomes despite receiving chemotherapy. The current study focused on patients treated primarily with radiation in order to maximally homogenize the cohort thus allowing us the opportunity to most accurately quantify the impact of tobacco exposure on survival. Clearly, given variable trends in surgery-based treatment for HPV + OPC, additional studies will be required to validate our findings in surgically treated cohorts.

In addition, the tobacco exposure range for the HPV+ cohort is substantially skewed toward the lower range of exposure (Supplementary Figure 2), likely limiting our ability to generate a more granular, dose-dependent effect for tobacco exposure on survival. Moreover, data suggests that current smoking status adversely affects LRC and OS in patients with HNSCC [39, 40]. This is further exacerbated by the smoking-induced reduction of radiation-induced tumor killing with subsequent worsening of locoregional control [41, 42]. The unavailability of smoking status for patients while on-treatment –as is the case with our study- might raise a question on the weight that should be assigned to the carcinogenic effect of smoking compared to its antagonistic impact on radiotherapy efficacy. Nonetheless, our results showed no differential disease control or overall survival between current and former smokers, even in the heavy smoker subset (PY > 30). We acknowledge that our study lacks a detailed categorization of history of tobacco exposure per the International Classification of Diseases, Tenth Revision (ICD-10) diagnosis code, as a result of inherent flaws of retrospective data collection [43]. However, it is still intuitive to extricate from our results the useful public health message relating to advice concerning smoking cessation, especially during radiotherapy course [44]. Benefits can include limiting radiotherapy/chemotherapy treatment prolongation or interruption and associated heavy symptom burden during and following treatment, in addition to the rapid return of carboxyhemoglobin levels in patients who quit to that of light /never smokers [45, 46].

Conversely, by limiting our analysis to only those patients with concordant p16 and HPV testing data, we can feel very confident that however limited the data, it is in fact reflective of the underlying tumor biology and not simply a testing artifact. The data generated here conform to what we have come to expect from OPSCC defined by conventional risk factor exposure (i.e. tobacco exposure), namely decreased treatment response and a high rate of loco-regional failure. We previously showed that > 90% of recurrence/progression occurs loco-regionally in a patient cohort with significant tobacco exposure [8]. The fact that tobacco exposure is a distinct competing risk for loco-regional failure not only suggests an impact on treatment effectiveness, but is particularly concerning when considering current efforts to de-escalate treatment for HPV+ OPSCC patients.

Based on this dataset, we strongly recommend development of a multi-institutional cooperative group focused on characterizing and quantifying the relative impact of tobacco exposure on HPV + OPSCC clinical outcomes. Until such time that definitive national datasets can be generated, we recommend strong consideration of tobacco exposure in the context of ongoing institutional and cooperative group trials aimed at de-escalation regimens for HPV+ OPSCC along the same lines as the phase II/III PATHOS and the recently completed NRG HN002 randomized controlled trials [47, 48]. Furthermore, we recommend a dedicated multi-institutional effort aimed at validating the current dataset and developing additional guidance for consideration of tobacco exposure in the context of the AJCC 8th Edition Staging Manual.

## Conclusion

Tobacco remains a critical driver of survival and treatment response in patients with HPV associated OPSCC receiving radiation treatment. HPV associated OPSCC in smokers should be considered a distinct entity after validation of this dataset in multi-institutional and prospective settings.

## Supplementary information

**Additional file 1: Supplementary Figure 1**. CONSORT flow diagram of selection process of patients for this study (OPC: oropharynx cancer; IMRT: intensity-modulated radiotherapy; HPV: human papillomavirus; ISH: in situ hybridization; IHC: immunohistochemistry).

**Additional file 2: Supplementary Figure 2**. Histogram of tobacco exposure in human papillomavirus-mediated (HPV+) and HPV- groups.

**Additional file 3: Supplementary Figure** 3 Parts A-B. Impact of human papillomavirus (HPV) status on survival. Kaplan-Meier plots for clinical outcomes for the entire patient cohort stratified by HPV status: (A) Loco-regional control; and (B) Freedom from distant metastasis.

**Additional file 4: Supplementary Figure 4** Parts A-B. Impact of tobacco exposure on survival. Kaplan-Meier plots for clinical outcomes for the HPV+ oropharyngeal cancer group stratified by extent of tobacco exposure: (A) Loco-regional control; and (B) Freedom from distant metastasis.

**Additional file 5: Supplementary Table 1**. HPV and tobacco impact on overall survival. Uni- and multi-variable analyses and corresponding hazard ratios and 95% confidence intervals (CI) for clinical variables associated with overall survival for the entire cohort stratified by human papillomavirus (HPV) status. *For AJCC stage (7th edition) analysis; stages I-III were collectively compared against stage IV (the majority class) given the imbalanced distribution of patients among AJCC 7th stages.

**Additional file 6: Supplementary Table 2**. HPV and tobacco impact on disease free survival. Uni- and multi-variable analyses and corresponding hazard ratios and 95% confidence intervals (CI) for clinical variables associated with disease-free survival for the entire patients cohort stratified by HPV status. *For AJCC stage (7th edition) analysis; stages I-III were collectively compared against stage IV (the majority class) given the imbalanced distribution of patients among AJCC 7th stages.

## Data Availability

Clinical dataset is not available as it includes personal health identifiers (PHI). It is possible for de-identified data to be made available upon reasonable request.

## Abbreviations

AJCC: American Joint Committee on Cancer
BIC: Bayesian information criteria
CCI: Charlson Comorbidity Index
CI: Confidence interval
DFS: Disease-free survival
EBRT: External beam radiotherapy
FDM: Freedom from distant metastasis
HPV: Human papillomavirus
HR: Hazard ratio
ICD-10: International Classification of Diseases, Tenth Revision
IHC: Immunohistochemistry
IMRT: Intensity-modulated radiotherapy
ISH: in situ hybridization
LRC: Loco-regional control
MACH-NC: Meta-analysis of Chemotherapy in Head and Neck Cancer
NRG: An organization that brings together the National Surgical Adjuvant Breast and Bowel Project (NSABP), the Radiation Therapy Oncology Group (RTOG), and the Gynecologic Oncology Group (GOG)
OPSCC: Oropharyngeal squamous cell carcinoma
OS: Overall survival
PY: Pack-years
RPA: Recursive partitioning analysis
US: United States
