# Supplementary figures and images for: Tobacco exposure as a major modifier of oncologic outcomes in human papillomavirus (HPV) associated oropharyngeal squamous cell carcinoma
Source: BMC Cancer. 2020 Sep 23;20:912. doi: 10.1186/s12885-020-07427-7 (PMC7513300; doi:10.1186/s12885-020-07427-7)

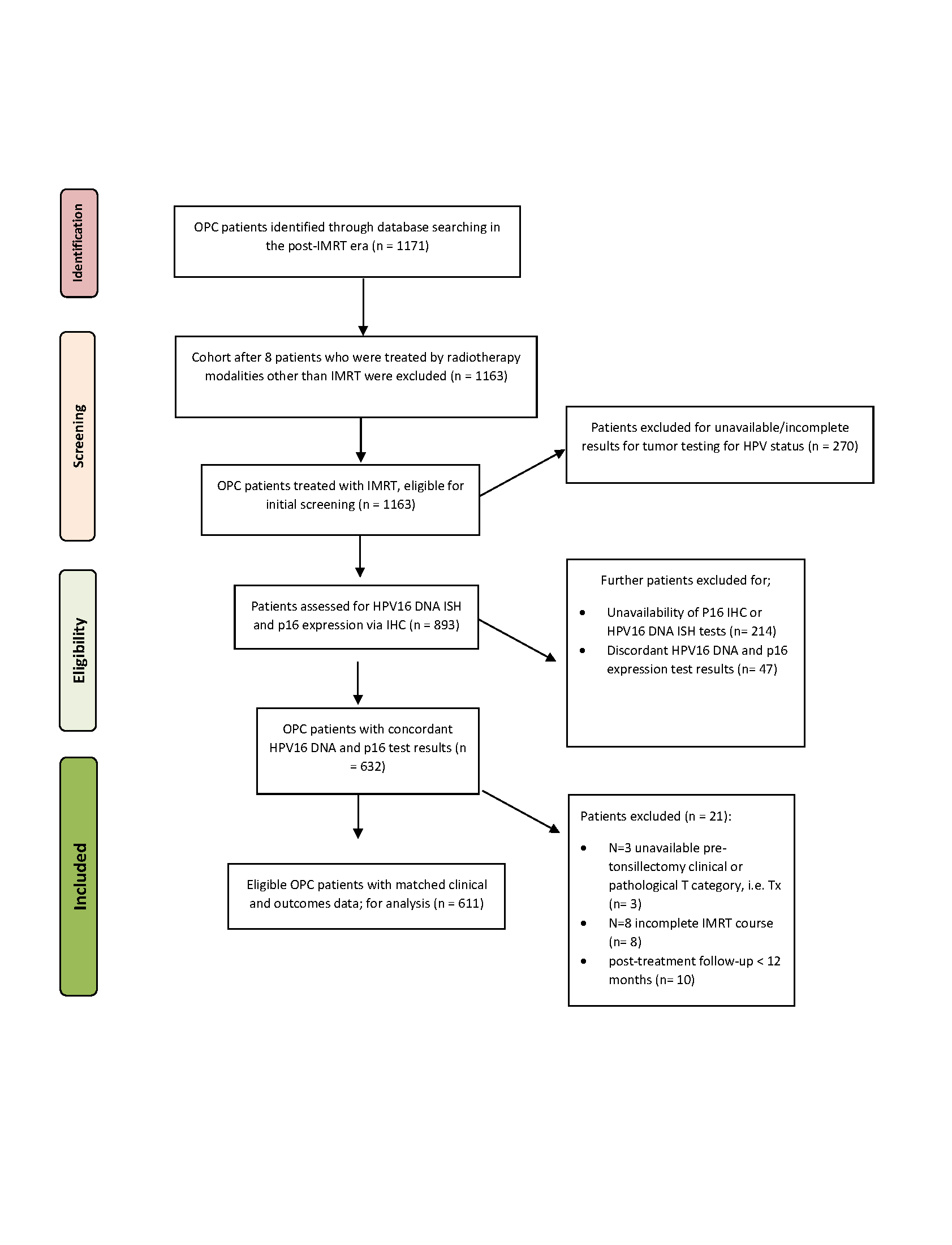

Supplement: Supplementary file 1 — Additional file 1: Supplementary Figure 1. CONSORT flow diagram of selection process of patients for this study (OPC: oropharynx cancer; IMRT: intensity-modulated radiotherapy; HPV: human papillomavirus; ISH: in situ hybridization; IHC: immunohistochemistry). [file 12885_2020_7427_MOESM1_ESM.png]

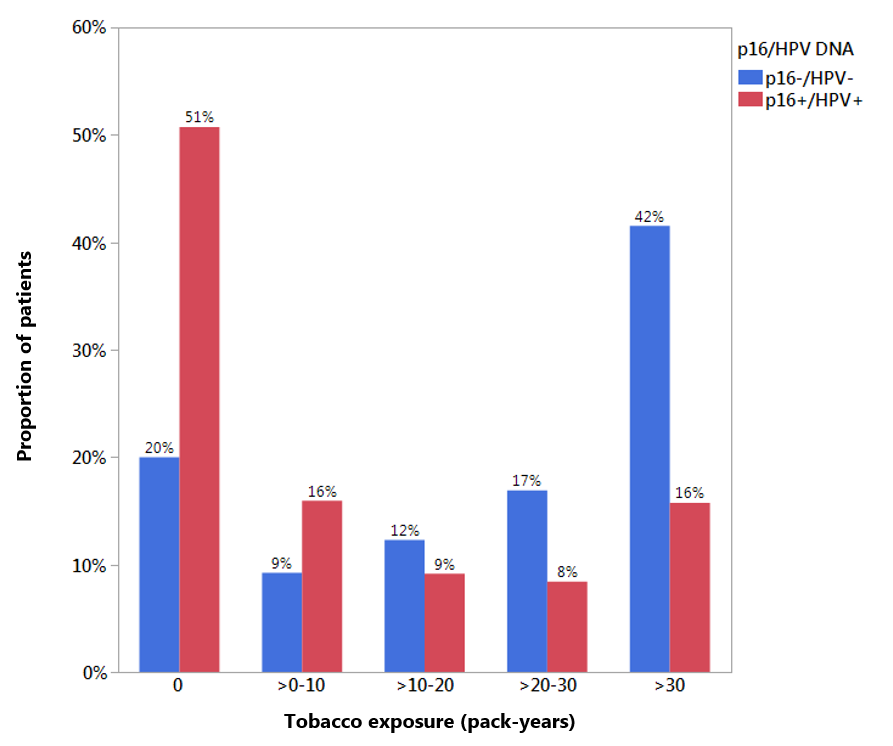

Supplement: Supplementary file 2 — Additional file 2: Supplementary Figure 2. Histogram of tobacco exposure in human papillomavirus-mediated (HPV+) and HPV- groups. [file 12885_2020_7427_MOESM2_ESM.png]

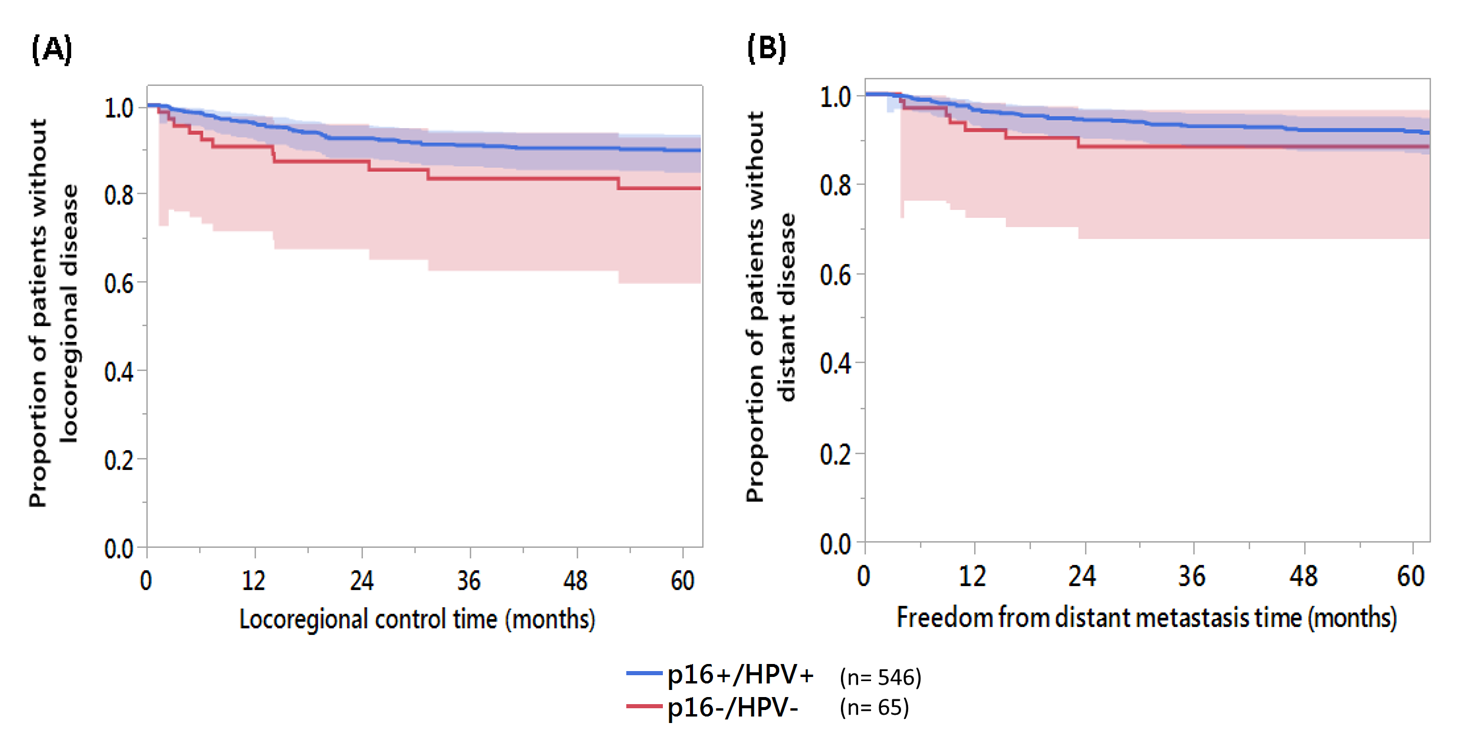

Supplement: Supplementary file 3 — Additional file 3: Supplementary Figure 3 Parts A-B. Impact of human papillomavirus (HPV) status on survival. Kaplan-Meier plots for clinical outcomes for the entire patient cohort stratified by HPV status: (A) Loco-regional control; and (B) Freedom from distant metastasis. [file 12885_2020_7427_MOESM3_ESM.tiff]

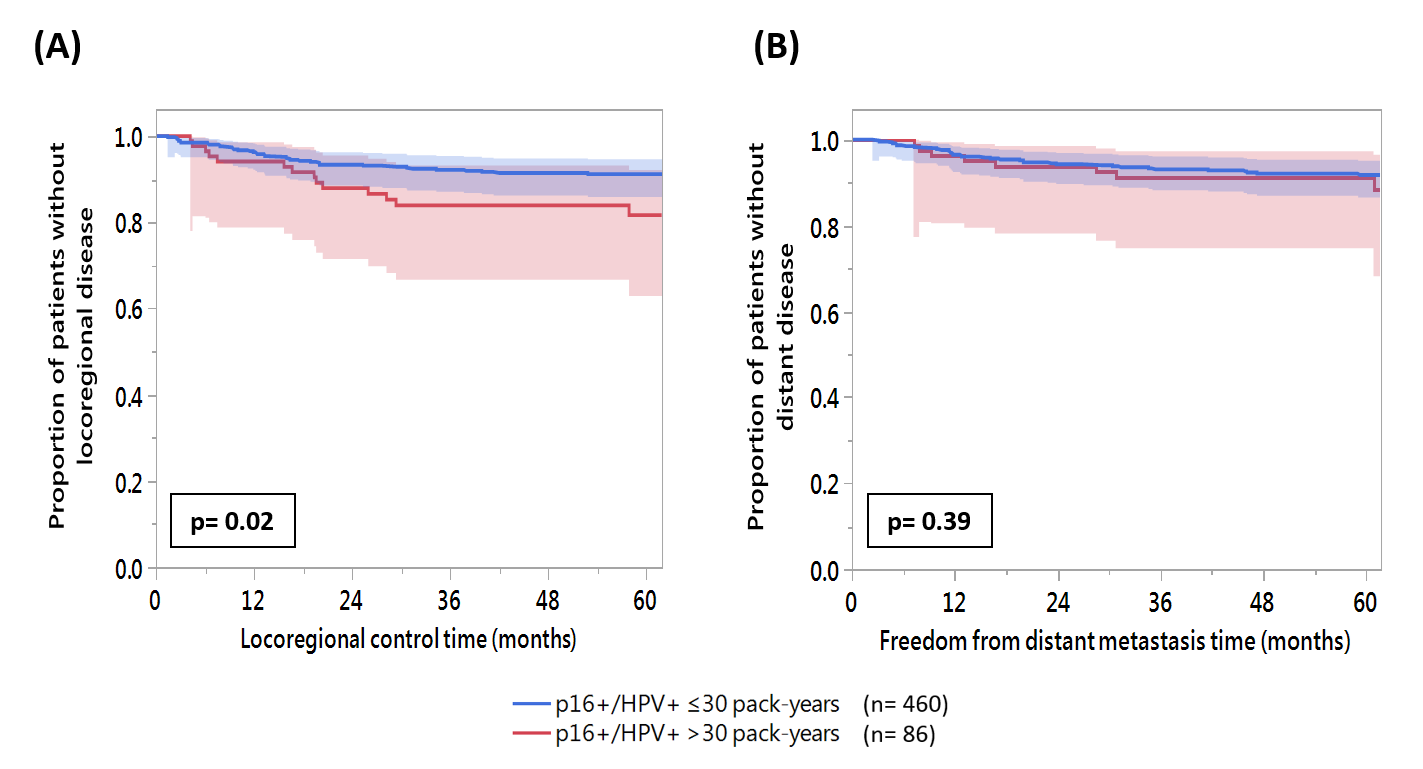

Supplement: Supplementary file 4 — Additional file 4: Supplementary Figure 4 Parts A-B. Impact of tobacco exposure on survival. Kaplan-Meier plots for clinical outcomes for the HPV+ oropharyngeal cancer group stratified by extent of tobacco exposure: (A) Loco-regional control; and (B) Freedom from distant metastasis. [file 12885_2020_7427_MOESM4_ESM.tiff]
