# Supplementary material for: Tobacco exposure as a major modifier of oncologic outcomes in human papillomavirus (HPV) associated oropharyngeal squamous cell carcinoma
Source: BMC Cancer. 2020 Sep 23;20:912. doi: 10.1186/s12885-020-07427-7 (PMC7513300; doi:10.1186/s12885-020-07427-7)
Supplement: Supplementary file 5 — Additional file 5: Supplementary Table 1. HPV and tobacco impact on overall survival. Uni- and multi-variable analyses and corresponding hazard ratios and 95% confidence intervals (CI) for clinical variables associated with overall survival for the entire cohort stratified by human papillomavirus (HPV) status. *For AJCC stage (7th edition) analysis; stages I-III were collectively compared against stage IV (the majority class) given the imbalanced distribution of patients among AJCC 7th stages. [file 12885_2020_7427_MOESM5_ESM.docx]

**Supplementary Table 1. HPV and tobacco impact on overall survival.** Uni- and multi-variable analyses and corresponding hazard ratios and 95% confidence intervals (CI) for clinical variables associated with overall survival for the entire cohort stratified by human papillomavirus (HPV) status. *For AJCC stage (7^th^ edition) analysis; stages I-III were collectively compared against stage IV (the majority class) given the imbalanced distribution of patients among AJCC 7^th^ stages

|  | HPV+ | | | | HPV- | | | |
| --- | --- | --- | --- | --- | --- | --- | --- | --- |
|  | Univariable analysis | | Multivariable analysis | | Univariable analysis | | Multivariable analysis | |
| Clinical variable | Hazard ratio (95% CI) | p-value | Hazard ratio (95% CI) | p-value | Hazard ratio (95% CI) | p-value | Hazard ratio (95% CI) | p-value |
| Age at diagnosis | __ | 0.160 | __ | 0.590 | __ | 0.210 | __ | 0.540 |
| Sex |  |  |  |  |  |  |  |  |
| Female | __ | __ | __ | __ | __ | __ | __ | __ |
| Male | 2.26 (1.01-6.45) | *0.047* | 1.94 (0.86-5.57) | 0.170 | 0.68 (0.27-1.84) | 0.420 | 1.24 (0.41-4.09) | 0.920 |
| CCI score  0  ≥1 | __  2.2 (1.3-3.5) | *0.003* | __  1.9 (1.1-3.1) | *0.010* | __  2.4 (0.9-6) | 0.090 | __  1.4 (0.45-4.4) | 0.500 |
| Chemotherapy (Cth) sequence |  |  |  |  |  |  |  |  |
| No Cth | __ | __ | __ | __ | __ | __ | __ | __ |
| IC only | 1.08 (0.35-3.24) | 0.900 | 0.8 (0.25-2.49) | 0.700 | 1.71 (0.32-9.24) | 0.51 | 1.97 (0.24-20.15) | 0.800 |
| CC only | 1.93 (0.89-4.81) | 0.100 | 1.21 (0.5-3.21) | 0.650 | 1.68 (0.5-7.6) | 0.42 | 1.26 (0.24-9.62) | 0.770 |
| IC + CC | 3.13 (1.47-7.73) | *0.002* | 1.29 (0.5-3.66) | 0.350 | 0.62 (0.11-3.32) | 0.55 | 0.31 (0.03-3.28) | 0.240 |
| AJCC stage (7^th^ edition) * |  |  |  |  |  |  |  |  |
| I-III | __ | __ | __ | __ | __ | __ | __ | __ |
| IV | 1.8 (0.92-4.06) | *0.090* | 1.3 (0.63-3.2) | 0.450 | 1 (0.38-3.13) | 1.000 | 2.11 (0.57-8.55) | 0.280 |
| AJCC stage (8^th^ edition) |  |  |  |  |  |  |  |  |
| I | __ | __ | __ | __ |  |  |  |  |
| II | 1.49 (0.83-2.61) | 0.180 | 1.07 (0.56-2.02) | 0.840 |  |  |  |  |
| III | 3.6 (2.08-6.18) | *<0.001* | 2.38 (1.23-4.68) | *0.011* |  |  |  |  |
| IV | __ | __ | __ | __ |  |  |  |  |
| T-category |  |  |  |  |  |  |  |  |
| T1 | __ | __ | __ | __ | __ | __ | __ | __ |
| T2 | 2.04 (1.04-4.29) | *0.040* | __ | __ | 3.31 (0.76-22.57) | 0.110 | __ | __ |
| T3 | 2.43 (1.12-5.42) | *0.020* | __ | __ | 2.77 (0.54-19.97) | 0.220 | __ | __ |
| T4 | 5.08 (2.48-11) | *<0.001* | __ | __ | 5.17 (1.18-35.4) | *0.030* | __ | __ |
| N-category (8^th^ edition) |  |  |  |  |  |  |  |  |
| N0 | __ | __ | __ | __ | __ | __ | __ | __ |
| N1 | 1.24 (0.5-4.12) | 0.670 | __ | __ | 0.71 (0.13-3.83) | 0.670 | __ | __ |
| N2 | 1.9 (0.72-6.53) | 0.210 | __ | __ | 0.55 (0.17-2.45) | 0.390 | __ | __ |
| N3 | 3.32 (0.78-14.05) | 0.100 | __ | __ | 1.7 (0.08-13.31) | 0.660 | __ | __ |
| Smoking (binary 30) |  |  |  |  |  |  |  |  |
| ≤30 | __ | __ | __ | __ | __ | __ | __ | __ |
| >30 | 2.58 (1.53-4.19) | *<0.001* | 1.97 (1. 16-3.25) | *0.010* | 2.74 (1.08-7.46) | *0.040* | 2.9 (0.95-9) | 0.059 |
| Total RT dose |  |  |  |  |  |  |  |  |
| 60-<70 | __ | __ | __ | __ | __ | __ | __ | __ |
| ≥70 | 2.49 (1.5-4.31) | *<0.001* | 1.53 (0.81-2.98) | 0.190 | 2.06 (0.78-6.44) | 0.150 | 2.9 (0.95-10.2) | 0.060 |
