# Supplementary material for: Tobacco exposure as a major modifier of oncologic outcomes in human papillomavirus (HPV) associated oropharyngeal squamous cell carcinoma
Source: BMC Cancer. 2020 Sep 23;20:912. doi: 10.1186/s12885-020-07427-7 (PMC7513300; doi:10.1186/s12885-020-07427-7)
Supplement: Supplementary file 6 — Additional file 6: Supplementary Table 2. HPV and tobacco impact on disease free survival. Uni- and multi-variable analyses and corresponding hazard ratios and 95% confidence intervals (CI) for clinical variables associated with disease-free survival for the entire patients cohort stratified by HPV status. *For AJCC stage (7th edition) analysis; stages I-III were collectively compared against stage IV (the majority class) given the imbalanced distribution of patients among AJCC 7th stages. [file 12885_2020_7427_MOESM6_ESM.docx]

**Supplementary Table 2. HPV and tobacco impact on disease free survival.**  Uni- and multi-variable analyses and corresponding hazard ratios and 95% confidence intervals (CI) for clinical variables associated with disease-free survival for the entire patients cohort stratified by HPV status. *For AJCC stage (7^th^ edition) analysis; stages I-III were collectively compared against stage IV (the majority class) given the imbalanced distribution of patients among AJCC 7^th^ stages

|  | HPV+ | | | | HPV- | | | |
| --- | --- | --- | --- | --- | --- | --- | --- | --- |
|  | Univariable analysis | | Multivariable analysis | | Univariable analysis | | Multivariable analysis | |
| Clinical variable | Hazard ratio (95% CI) | p-value | Hazard ratio (95% CI) | p-value | Hazard ratio (95% CI) | p-value | Hazard ratio (95% CI) | p-value |
| Age at diagnosis | __ | 0.4 | __ | 0.57 | __ | *0.04* | __ | 0.25 |
| Sex |  |  |  |  |  |  |  |  |
| Female | __ | __ | __ | __ | __ | __ | __ | __ |
| Male | 1.71 (0.85-4.1) | 0.14 | 1.51 (0.74-3.62) | 0.28 | 1.19 (0.45-3.7) | 0.74 | 1.48 (0.51-5.08) | 0.48 |
| Chemotherapy (Cth) sequence |  |  |  |  |  |  |  |  |
| No Cth | __ | __ | __ | __ | __ | __ | __ | __ |
| IC only | 1.41 (0.51-4.03) | 0.5 | 1.16 (0.41-3.4) | 1.16 | 0.33 (0.02-2.02) | 0.25 | 0.16 (0.007-1.52) | 0.12 |
| CC only | 2.3 (1.08-5.66) | *0.03* | 1.71 (0.74-4.43) | 0.22 | 0.82 (0.26-2.77) | 0.73 | 0.29 (0.06-1.37) | 0.12 |
| IC + CC | 3.34 (1.57-8.22) | *0.001* | 1.87 (0.75-5.17) | 0.18 | 0.65 (1.8-2.33) | 0.49 | 0.16 (0.02-1) | 0.05 |
| AJCC stage (7^th^ edition)* |  |  |  |  |  |  |  |  |
| I-III | __ | __ | __ | __ | __ | __ | __ | __ |
| IV | 1.56 (0.85-3.23) | 0.16 | 1.14 (0.57-2.48) | 0.72 | 1.01 (0.38-3.14) | 0.99 | __ | __ |
| AJCC stage (8^th^ edition) |  |  |  |  |  |  |  |  |
| I | __ | __ | __ | __ |  |  |  |  |
| II | 1.46 (0.86-2.44) | 0.16 | 1.09 (0.61-1.96) | 0.76 |  |  |  |  |
| III | 2.9 (1.69-4.88) | *0.0002* | 1.98 (1.04-3.75) | *0.04* |  |  |  |  |
| IV | __ | __ | __ | __ |  |  |  |  |
| T-category |  |  |  |  |  |  |  |  |
| T1 | __ | __ | __ | __ | __ | __ | __ | __ |
| T2 | 2.37 (1.26-4.77) | *0.007* | __ | __ | 9.41 (1.72-174.71) | *0.007* | __ | __ |
| T3 | 2.44 (1.17-5.25) | *0.02* | __ | __ | 7.68 (1.24-147.37) | *0.03* | __ | __ |
| T4 | 4.55 (2.24-9.65) | *<0.0001* | __ | __ | 7.24 (1.07-141.88) | *0.04* | __ | __ |
| N-category (8^th^ edition) |  |  |  |  |  |  |  |  |
| N0 | __ | __ | __ | __ | __ | __ | __ | __ |
| N1 | 1.8 (0.66-7.42) | 0.28 | __ | __ | 1.67 (0.33-12.04) | 0.55 | __ | __ |
| N2 | 2.98 (1.05-12.52) | *0.04* | __ | __ | 0.92 (0.25-5.92) | 0.91 | __ | __ |
| N3 | 5.37 (1.31-26.32) | *0.02* | __ | __ | 2.94 (0.14-30.81) | 0.41 | __ | __ |
| Smoking (binary 30) |  |  |  |  |  |  |  |  |
| ≤30 | __ | __ | __ | __ | __ | __ | __ | __ |
| >30 | 1.63 (0.94-2.68) | 0.09 | 1.36 (0.78-2.27) | 0.27 | 2.24 (0.88-5.87) | 0.09 | 2.74 (0.95-8.26) | 0.06 |
| Total RT dose |  |  |  |  |  |  |  |  |
| 60-<70 | __ | __ | __ | __ | __ | __ | __ | __ |
| ≥70 | 2.06 (1.3-3.39) | *0.002* | 1.28 (0.72-2.34) | 0.4 | 2.94 (1.05-10.41) | *0.04* | 5.45 (1.54-25.09) | *0.007* |
